# Targeting PI3K Pathway in Pancreatic Ductal Adenocarcinoma: Rationale and Progress

**DOI:** 10.3390/cancers13174434

**Published:** 2021-09-02

**Authors:** Siddharth Mehra, Nilesh Deshpande, Nagaraj Nagathihalli

**Affiliations:** 1 Division of Surgical Oncology, Department of Surgery, Miller School of Medicine, University of Miami, Miami, FL 33136, USA; sxm1884@med.miami.edu (S.M.); nud1@miami.edu (N.D.); 2 Sylvester Comprehensive Cancer Center, University of Miami, Miami, FL 33136, USA

**Keywords:** pancreatic cancer, PI3K/Akt pathway, RAS, Urolithin A, tumor-stromal crosstalk, immune microenvironment

## Abstract

**Simple Summary:**

Pancreatic cancer has a dismal 5-year survival rate of 10%, making it one of the deadliest forms of malignancy. The poor prognosis associated with this disease is because it is resistant to almost every type of chemotherapy. Another major hallmark of pancreatic cancer is the presence of several activated oncogenic signaling pathways, including PI3K/Akt/mTOR, which promotes disease aggressiveness and therapeutic resistance. Previously, we have shown that targeted inhibition of PI3K/Akt/mTOR together led to a significant reduction of tumor burden and improvement of overall survival in an aggressive mouse model of pancreatic cancer. This review article discusses the significance of targeting PI3K and its direct downstream effector components in pancreatic cancer. Additionally, we will also update on the recent studies highlighting the tumor cell-extrinsic impact of PI3K inhibition in the modulation of the immune microenvironment within the context of this malignancy.

**Abstract:**

Pancreatic ductal adenocarcinoma (PDAC) remains among the deadliest solid tumors that remain treatment-refractory and show a dismal prognosis. More than 90% of PDAC tumors harbor mutations in the K-Ras that exert a strong pro-tumorigenic effect by activating several downstream effector pathways, including phosphatidylinositol-3-kinase (PI3K)-Akt. The role of frequently activated PI3K/Akt pathway in promoting PDAC aggressiveness is well established. Therapeutic approaches targeting PI3K and downstream signaling components in different cellular compartments, including tumor, stromal and immune cells, have directly impacted the tumor burden in this cancer type. Our previous work has demonstrated that targeting the PI3K/Akt/mTOR pathway reduced tumor growth and improved survival in the genetic mouse model of PDAC. Here, we discuss the significance of targeting PI3K signaling and the biological impact of PI3K inhibition in modulating the tumor–stromal immune crosstalk within the microenvironment of pancreatic cancer. Furthermore, this review updates on the current challenges involving the therapeutic implications of targeting this pathway in PDAC.

## 1. Introduction

Pancreatic ductal adenocarcinoma (PDAC) is a highly aggressive malignancy emerging as one of the most frequent causes of cancer-related deaths globally [1,2,3]. With a 5-year survival rate of around 10%, the dismal prognosis associated with this disease is mainly attributed to its advanced clinical manifestation in most patients. Surgical resection is the only possible curative measure available in only 10–20% of patients diagnosed with this malignancy. For patients not eligible for immediate surgical resection, neoadjuvant chemotherapy is given to reduce the tumor burden before surgery [4,5,6]. Despite the recent advances in surgical care and chemotherapy regimens, only a modest improvement in survival has been observed with this disease. This suggests a clear need to improve our understanding of the biology of PDAC to facilitate early diagnosis and identify new molecular targets for a better therapeutic response to this malignancy.

**Figure 1:**
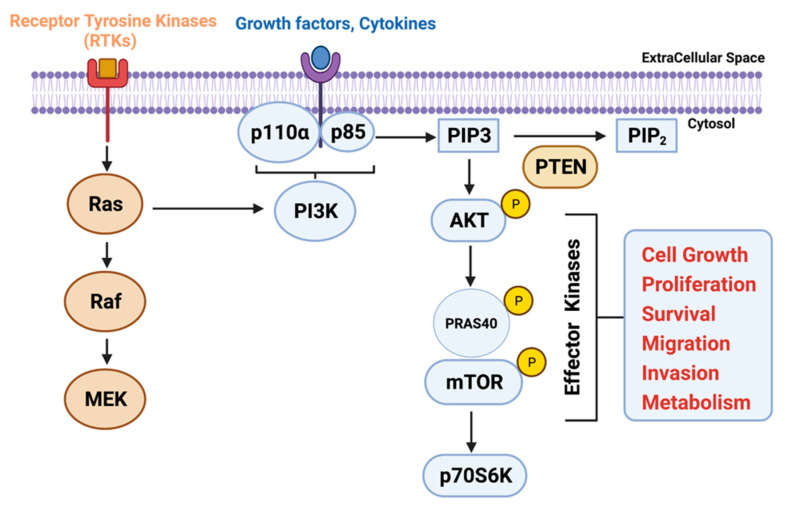
Schematic representation of PI3K/AKT/mTOR signaling pathway and its implication on cellular processes. The image was created with BioRender.com (Agreement number ZS22VAMSID, accessed on 23 August 2021).

One of the characteristic hallmarks of PDAC is the presence of the activating K-Ras mutation in more than 90% of patients with this disease [7,8]. This key oncogenic driver mutation is the earliest genetic alteration that is associated with the progression of this disease from early premalignant to invasive carcinoma [9]. The presence of mutant K-Ras regulates a myriad of downstream signaling pathways involved in several oncogenic processes. One such signaling pathway is phosphatidylinositol 3-kinase (PI3K) [10]. Analyzing The Cancer Genome Atlas (TCGA) database of 32 cancer types revealed the involvement of PI3K and its downstream signaling components in mediating the pro-tumorigenic effect of K-Ras and other oncogenes in numerous human malignancies, including PDAC [11,12]. Other than K-Ras-mediated activation of this cascade, PI3Ks pick up cues from growth factor stimuli and cytokines through receptor tyrosine kinases present on the cancer cells and send them to intracellular signals, controlling diverse oncogenic functions (Figure 1) [13]. The intricated network of PI3K signaling also acts as a central node for activating mTOR, NF-kB, GSK3ß, p27, and Bad-Bax pathways, which are known to regulate various aspects of cancer cell survival, growth, motility, and metabolism [14].

As in many cancer types, PDAC has also been a candidate for targeting the PI3K, including its downstream effectors, such as Akt and mTOR (mammalian Target of rapamycin). While PI3K inhibitors alone have shown limited success in treating PDAC patients, the use of PI3K inhibitors in combination with other drugs has shown promising results in pre-clinical studies. Previously, we have demonstrated that Urolithin A mediated simultaneous targeting of the PI3K/Akt/mTOR pathway reduced PDAC tumor growth and improved survival in the genetically engineered mouse model (GEMM) of PDAC [15]. In the current review, we discuss the recent progress that has been made in targeting PI3K and its key effector components. Additionally, this article will highlight the impact of PI3K inhibitors in modulating the tumor–stromal immune crosstalk within the context of this tumor type and current challenges or limitations of targeting the PI3K pathway in PDAC.

## 2. PI3K Signaling Activation in PDAC

An increase in the activation of the PI3K signaling is associated with poor overall survival in PDAC patients [16,17]. Other than the K-Ras-mediated activation of this signaling pathway, the presence of the oncogenic mutation in the PI3KCA gene itself is one of the known mechanisms of its hyperactivation; however, this mutation is found only in a few subsets of PDAC patients [7]. Examining the role of PI3KCA mutations in pancreatic tumorigenesis in a mouse model, Payne et al. demonstrated increased acinar-to-ductal metaplasia (ADM) and pancreatic intraepithelial neoplasms (PanINs) towards invasive PDAC in mice constitutively expressing this mutant form within their pancreas [18]. PDAC heavily relies on mutant K-Ras to activate a plethora of downstream signaling pathways driving pancreatic tumorigenesis. Attempts to target critical Ras effectors revealed a heavy dependency of K-Ras on the PI3K signaling cascade in humans as well as in several K-Ras driven GEMMs of PDAC [16,19,20]. Within the context of mutant K-Ras, overexpressing mutant PI3KCA (H1047R) drives PI3K-3-phosphoinositide-dependent protein kinase 1 (PDK1) signaling-mediated irreversible ADM reprogramming towards PDAC formation [19]. Additionally, Baer et al. further highlighted the importance of blocking specific class I p110alpha PI3K isoform in the presence of mutant K-Ras. Pancreas-specific inhibition of this isoform of PI3K significantly attenuated the transition of exocrine acinar cells towards pancreatic preneoplastic ductal lesions after pancreatic injury. Surprisingly, targeting the p110β isoform of PI3K did not prevent this preneoplastic transformation with mutant K-Ras [21].

Another critical molecule in the PI3K signaling node is a serine-threonine kinase Akt that belongs to the AGC kinase family [22]. Upon receiving the activating stimulus via PI3K, Akt is recruited to the plasma membrane by interacting the pleckstrin homology domain with membrane lipids [23]. PDK1 phosphorylates the recruited Akt at residues Thr308 and causes its activation [24,25]. Once activated, Akt regulates cell growth, proliferation, and survival by phosphorylating a variety of downstream antiapoptotic and cell-cycle-related proteins as well as transcription factors (Figure 1) [26]. Increased activity of Akt due to hyperphosphorylation events has been recorded in around 60% of PDAC samples, whereas its overexpression due to gene amplification was observed in 10–20% of PDAC patients [12,27,28]. 

Additional downstream effector players of the PI3K-Akt-signaling axis is a mammalian target of rapamycin (mTOR). mTOR is a serine/threonine kinase that participates in cell survival, growth, and apoptosis regulation. They usually exist as two complexes, rapamycin-sensitive is mTORC1 and rapamycin-insensitive is mTORC2. Akt is known to directly activate mTORC1 through phosphorylation of pRAS40, a vital component of the mTORC1 complex [29]. PI3K-dependent activation of the mTORC2 complex is also mediated via increased AKT phosphorylation [30]. These mTORs enhance the translational ability of several mRNAs in combination with other accessory protein complexes [31,32]. Several studies have established activation of the mTOR pathway in pancreatic cell lines, xenografts, and in human PDAC patients, suggesting the role of using rapamycin or rapalogs in this malignancy [33,34,35].

One of the major negative regulators of PI3K signaling is PTEN. It acts as a tumor suppressor and a natural antagonist of PI3K that relieves the repression of the PI3K/Akt signaling axis. Investigators have previously shown that in a Pdx1^CRE^ mouse model, conditional deletion of PTEN with mutant K-Ras within the pancreas accelerates the development of premalignant neoplasm via promoting ADM formation [36,37].

## 3. Inhibition of PI3K Signaling in PDAC

Considering the activation of PI3K and its crucial signaling effector components in this cancer type, intense research has been directed towards targeted inhibition of this pathway in several pre-clinical studies in PDAC. Along these lines, Bondar et al. reported dose-dependent induction of apoptosis in human PDAC cells treated with pan PI3K inhibitors Wortmannin and LY294002. Moreover, using an orthotopic mouse model, they further showed decreased tumor growth and metastases with increased tumor cell death in mice treated with the inhibitor LY294002 [38]. Another study by Schneider et al. demonstrated the role of a single therapeutic agent LY294002, a specific PI3K inhibitor, in sensitizing human PDAC cells to apoptosis in combination with non-steroidal anti-inflammatory drugs (NSAIDS) [39].

Over the years, several classes of small molecule inhibitors targeting PI3K signaling nodes have shown promising therapeutic efficacy in cancers, including breast, ovarian, and NSCLC [40]. However, despite the success of many of these drugs in other tumor types, patients with PDAC have shown suboptimal responses to monotherapies against these PI3K signaling inhibitors. Thus, to overcome the limitations associated with monotherapy regimens for PDAC treatment, the focus has recently shifted to employ combinatorial approaches. To better arrest the tumor progression in PDAC, efforts have been made to use PI3K inhibitors to combine small molecule attenuators of its downstream effector pathways.

**Figure 2:**
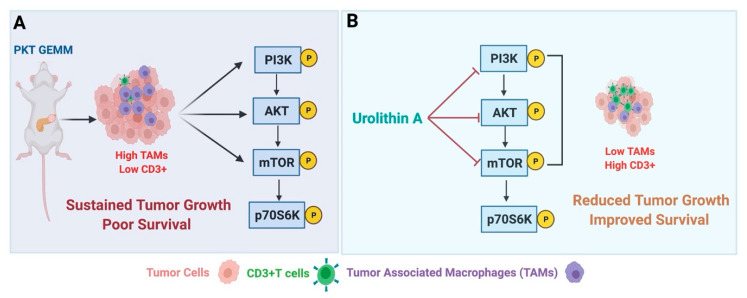
Schematic representation showing the effect of Uro A in a genetically engineered mouse model (GEMM) of PDAC. (**A**) Ptf1a^Cre/+^; LSL-Kras^G12D/+^; Tgfbr2^flox/flox^ (PKT) GEMM showing sustained pancreatic tumor growth, presence of immunosuppressive tumor-associated macrophages (TAMs), lack of CD3+T cells, and activated PI3K/AKT/mTOR signaling axis within the microenvironment of a highly aggressive Ptf1a^Cre/+^; LSL-Kras^G12D/+^; Tgfbr2^flox/flox^ (PKT) genetically engineered mouse model (GEMM) of PDAC. (**B**) Treatment with Urolithin A (Uro A) intercedes its anti-tumor effects by targeting PI3K/AKT/mTOR kinase pathways to overcome immunosuppressive tumor microenvironment, thereby reducing TAMs and increases CD3+T cells. PKT mice treated with Uro A displayed reduced tumor growth and marked improvement in overall survival compared to vehicle-treated mice. Figure adapted from Totiger et al. [15] The image was created with BioRender.com (Agreement number ZS22VAMSID, accessed on 23 August 2021).

The therapeutic efficacy of MK2206 alone (an allosteric pan Akt inhibitor) in PDAC was associated with a modest anti-tumor response in patients with this malignancy [41]. Furthermore, treatment with this Akt inhibitor alone (MK2206) displayed no significant change in the overall survival in an aggressive Ptf1a^Cre/+^; LSL-Kras^G12D/+^; Tgfbr2^flox/flox^ (PKT) GEMM of PDAC that recapitulates the human disease regarding dense desmoplastic stroma and aggressiveness (unpublished data). As opposed to the suboptimal response of this inhibitor when used as a monotherapy in PDAC, Hu et al. reported dramatic anti-tumor effects of MK2206 when used in combination with CDK inhibitor dinaciclib (MK-7965) in several pre-clinical mouse models of PDAC [42]. Another possible mechanism of therapeutic resistance against PI3K inhibitors was shown to be mediated by the systemic glucose–insulin feedback loop involved in the reactivation of PI3K/mTOR signaling axis [43]. Targeting the mTORC1 activity loop alone has proven to be ineffective, mainly due to increased activation of the PI3K axis and loss of mTORp70S6K-negative feedback [44]. Therefore, targeting dual mTOR and PI3K is vital to avoid pathway reactivation. In this regard, Cao et al. reported a significant reduction in tumor burden, and decreased phosphorylation levels of PI3K and Akt in the orthotopic mouse model of PDAC with NVP-BEZ235 dual-class I PI3K/mTOR inhibitor [45]. In one of the crucial findings from our lab, we have identified Urolithin A, a natural compound capable of simultaneously targeting both PI3K/Akt and mTOR. Therapeutic inhibition of this pathway node by Urolithin A reduced tumor growth, proliferation, and migration in vitro and significantly improved overall survival in highly aggressive PKT GEMM of PDAC (Figure 2) [15]. 

## 4. Reciprocal Crosstalk Involving PI3K Signaling in PDAC

One of the primary reasons for the failure of targeted therapy in mutant K-Ras mediated tumorigenesis in PDAC is the activation of multiple downstream effectors signaling cascades [46]. The extensive crosstalk between the two most redundant PI3K/Akt/mTOR and mitogen-activated protein kinase (MAPK) pathways appears to be clinically relevant, and it might be a potential reason for a poor therapeutic response associated with PI3K inhibitors alone in PDAC. Within this context, Wong et al. demonstrated the increased therapeutic efficacy of the dual combined blockade of MAPK and PI3K/Akt/mTOR to reduce oncogenic potential and overcome therapeutic resistance in human PDAC cell lines [47]. High-throughput screening of 46 PDAC cell lines for clinically relevant therapeutic agents by Alageson et al. showed that most PDAC cell lines resist single-agent therapies. Treatment with MEKi AZD6244 alone was mainly cytostatic. However, tumor cell apoptosis was maximized only when combined with PI3K inhibitors (BKM120 or GDC-0941). This combination treatment also delayed tumor formation and extended overall survival in GEMM of PDAC [48]. To model these findings in actual clinical settings, Juntilla et al. further reported an incrementally enhanced overall survival with triple combination therapy of gemcitabine with MEK and PI3K inhibition compared to gemcitabine alone [49]. Another plausible mechanism of adaptive resistance against therapies targeting mutant K-Ras or MEK in PDAC is the activation of integrin-linked kinase (ILK)-mediated increased phosphorylation of the mTORC2 component Rictor and Akt. Only co-targeting mTORC1/2 with mutant K-Ras or MEK led to a durable and sustained anti-tumor response in PDAC compared to any of the monotherapies [50]. In summary, compensatory activation of alternate signaling pathways involving feedforward loops acts as a potential resistance mechanism that leads to the recurrence of tumors, limiting therapeutic response with monotherapies targeting PI3K signaling components in PDAC.

## 5. Impact of PI3K Inhibition on Tumor–Stromal Immune Crosstalk in PDAC

The PDAC tumor microenvironment (TME) is characterized by a dense stromal network comprised of non-neoplastic cells, extracellular matrix components, and the presence of immunosuppressive cells constituting mainly myeloid-derived suppressor cells (MDSCs), tumor-associated macrophages (TAMs), and regulatory T cells [51,52,53]. The dynamicity of TME in PDAC is mediated by crosstalk between these cell types through paracrine mediators, including growth factors and cytokines that result in tumor cell proliferation, chemoresistance, metastasis, and altered apoptotic potential [54]. Interestingly, PI3K and its downstream cascade players are also involved in modulating of this tumor–stromal–immune crosstalk, thereby facilitating tumor progression and promoting therapeutic resistance in PDAC [55].

Cancer-associated fibroblasts (CAFs) constitute one of the significant stromal cell populations present in the TME of PDAC. They are master secretors of factors promoting chemoresistance in this malignancy. Duluc et al. reported the selective inhibition of CAF protein synthesis by blocking the mTOR/4E/BP1 and revealed that this pathway significantly alters their secretome profile and reduces stromal fibrosis to promote the therapeutic efficacy of gemcitabine in a GEMM of PDAC [56]. Although the direct action of pan PI3K or isoform-specific PI3K inhibitors on stromal fibroblasts has not yet been tested in PDAC, modulation of PI3K signaling could likely impact several aspects of tumor–stroma interaction and give us a more robust rationale to target this signaling node in PDAC in future years.

Other than the contribution of fibroinflammatory desmoplastic stroma in promoting innate and acquired resistance to several conventional therapeutic approaches in PDAC, the presence of cellular elements of innate immune populations, including TAMs and MDSCs, dampen the T cell infiltration and their activation [51,52,53]. Therefore, a significant unmet need in the field is identifying different therapeutic strategies that can remodel the immunologically cold PDAC TME to reactivate the anti-tumor immune responses. Along similar lines, using a highly aggressive PKT GEMM mouse of PDAC that phenocopies human disease in terms of dense stroma and presence of immunosuppressive milieu [57], previously we have demonstrated that combined inhibition of PI3K/Akt/mTOR using Urolithin A effectively reprograms the fibroinflammatory tumor stroma to promote an anti-tumor immune microenvironment by decreasing immunosuppressive TAMs and augmenting T-cell recruitment within the TME of PDAC (Figure 2) [15]. Importantly, treatment with pan AKT inhibitor MK-2206 did not affect any of these immune subsets in our mouse model, further highlighting the significance of targeting multiple nodes of this signaling axis in PDAC (unpublished data).

Tumor intrinsic activation of PI3K signaling, particularly PI3Kalpha, is associated with an inflammatory metastatic phenotype and leads to enhanced accumulation of protumorigenic M2 (CD206+) like macrophages in PDAC [17]. Reprogramming of TAMs towards an anti-tumorigenic M1 phenotype has been associated with increased immunogenicity and increased T cell infiltration within the TME of PDAC. In view of this, Li et al. demonstrated that TAM-specific inhibition of PI3Kγ using a nano micelle-targeted delivery system in combination with siRNA against colony-stimulating factor-1 receptor (CSF/CSF1R) pathways significantly reprogrammed the TAMs towards an M1-like antitumorigenic state. This resulted in an enhanced anti-tumor immune response in the mouse model of pancreatic cancer [58]. Overall, their findings reveal the critical role of isoform-specific inhibition of PI3K in immune cell compartment rather than just a tumor-intrinsic role in this malignancy.

Different isoforms of PI3K, including PI3Kγ and PI3Kδ, play diverse roles within specific immune cell compartments, and modulating their expression within these cells significantly alters the tumor burden and boosts the anti-tumor immune network of PDAC [59]. Kaneda et al. highlighted the role of myeloid cell specific PI3Kγ isoforms in promoting immunosuppressive transcriptional gene signatures within the macrophages, fueling tumor growth and desmoplasia in PDAC. Inhibition of PI3Kγ in PDAC-bearing mice reprograms the immunosuppressive TAM subsets to promote T cell-mediated anti-tumor immune response and led to a significant reduction in PDAC metastasis as well as desmoplasia, highlighting a new therapeutic modality for this devastating disease [60]. Targeted inhibition of PI3Kδ within the regulatory T cell (Tregs) compartment using the inhibitor PI-3065 significantly increased the cytolytic T cell infiltration (CD8+ T cells). It attenuated the tumor growth and prolonged survival in the GEMM of PDAC [59]. Studies over the years have been focused on understanding the role of PI3K signaling through the lens of AKT as a dominant effector molecule of this pathway. However, recently, AKT-independent signaling branches downstream of PI3K have been shown to play essential roles in promoting cancer-related phenotypes [61]. One such crucial signaling is the TEC family kinase BTK (Bruton’s tyrosine kinase), which acts downstream of PI3K and is involved in B cell receptor (BCR)-dependent cell proliferation. The significance of the PI3Kγ-BTK signaling axis in promoting macrophage CD4+Thelper2 (Th2) was shown to be associated with immune suppression in PDAC progression [62]. Additionally, targeted inhibition of BTK delayed PDAC tumor growth and provided enhanced anti-tumor immunity within the TME of this malignancy. These complex pre-clinical data imply that the role of PI3K and its isoforms in distinct cell types within the tumor microenvironment can contribute in a nuanced way to the overall tumor immune microenvironment and therapeutic responses.

## 6. Conclusions and Future Perspectives

Several pre-clinical and clinical assessments of targeted therapies in PDAC show that most of these tumors display inherent or acquired resistance. Unlike most solid tumors, the TME of PDAC consists of a highly complex mixture of cancer cells, immune cell population, and stromal cells. The dynamicity of this TME is maintained by regular crosstalk among these cell types, leading to tumor aggressiveness. Since the complexity of the mechanism governing this crosstalk is vast, combination therapies targeting these cell populations are essential in this malignancy to achieve a durable anti-tumor response. The network of signaling pathways regulated by PI3K identifies dynamic cues from the TME to either promote a multitude of oncogenic processes directly or activate a parallel interconnected signaling node. Moving forward with the advancements in targeting this pathway and understanding the molecular mechanisms of PI3K inhibitor unresponsiveness and resistance, better patient stratification using diagnostic biomarker-based study, defining PDAC subtypes based on cancer cells and the stromal component will be essential to design personalized therapies to achieve better clinical outcomes in PDAC.
